# *De novo* mutations, genetic mosaicism and human disease

**DOI:** 10.3389/fgene.2022.983668

**Published:** 2022-09-26

**Authors:** Mohiuddin Mohiuddin, R. Frank Kooy, Christopher E. Pearson

**Affiliations:** ^1^ Program of Genetics and Genome Biology, The Hospital for Sick Children, Toronto, ON, Canada; ^2^ Department of Medical Genetics, University of Antwerp, Edegem, Belgium; ^3^ Department of Molecular Genetics, University of Toronto, Toronto, ON, Canada

**Keywords:** *de novo* mutation, mosaicism, timing of mutation, repeat instability, germline mutation, somatic mutation, autism spectrum disorder, genetic diseases

## Abstract

Mosaicism—the existence of genetically distinct populations of cells in a particular organism—is an important cause of genetic disease. Mosaicism can appear as *de novo* DNA mutations, epigenetic alterations of DNA, and chromosomal abnormalities. Neurodevelopmental or neuropsychiatric diseases, including autism—often arise by *de novo* mutations that usually not present in either of the parents. *De novo* mutations might occur as early as in the parental germline, during embryonic, fetal development, and/or post-natally, through ageing and life. Mutation timing could lead to mutation burden of less than heterozygosity to approaching homozygosity. Developmental timing of somatic mutation attainment will affect the mutation load and distribution throughout the body. In this review, we discuss the timing of *de novo* mutations, spanning from mutations in the germ lineage (all ages), to post-zygotic, embryonic, fetal, and post-natal events, through aging to death. These factors can determine the tissue specific distribution and load of *de novo* mutations, which can affect disease. The disease threshold burden of somatic *de novo* mutations of a particular gene in any tissue will be important to define.

## Mosaicism - From germline to somatic

The term “mosaic” refers to an intricate image or pattern created by craftsmen from small pieces of colored hard material, such as glass, gems, ornamental stones or other precious material. At a distance, the collective image seems as it would in a painting. Only on close assessment, the individual components become distinguishably different. In biology, mosaicism means the presence of genetically different cells within a tissue of single organism (Youssoufian and Pyeritz, 2002; Biesecker and Spinner, 2013). An individual who has developed from a single fertilized egg and has two or more populations of cells with different genotypes is mosaic. In terms of the whole organism, rise of the mosaic phenotype depends on tissue-to-tissue genetic variations that do not conform to Mendelian rules of inheritance. During the last several years, germline and somatic mosaicism have appeared as critical factors that contribute to phenotypic variability in almost every area of biology.

**FIGURE 1:**
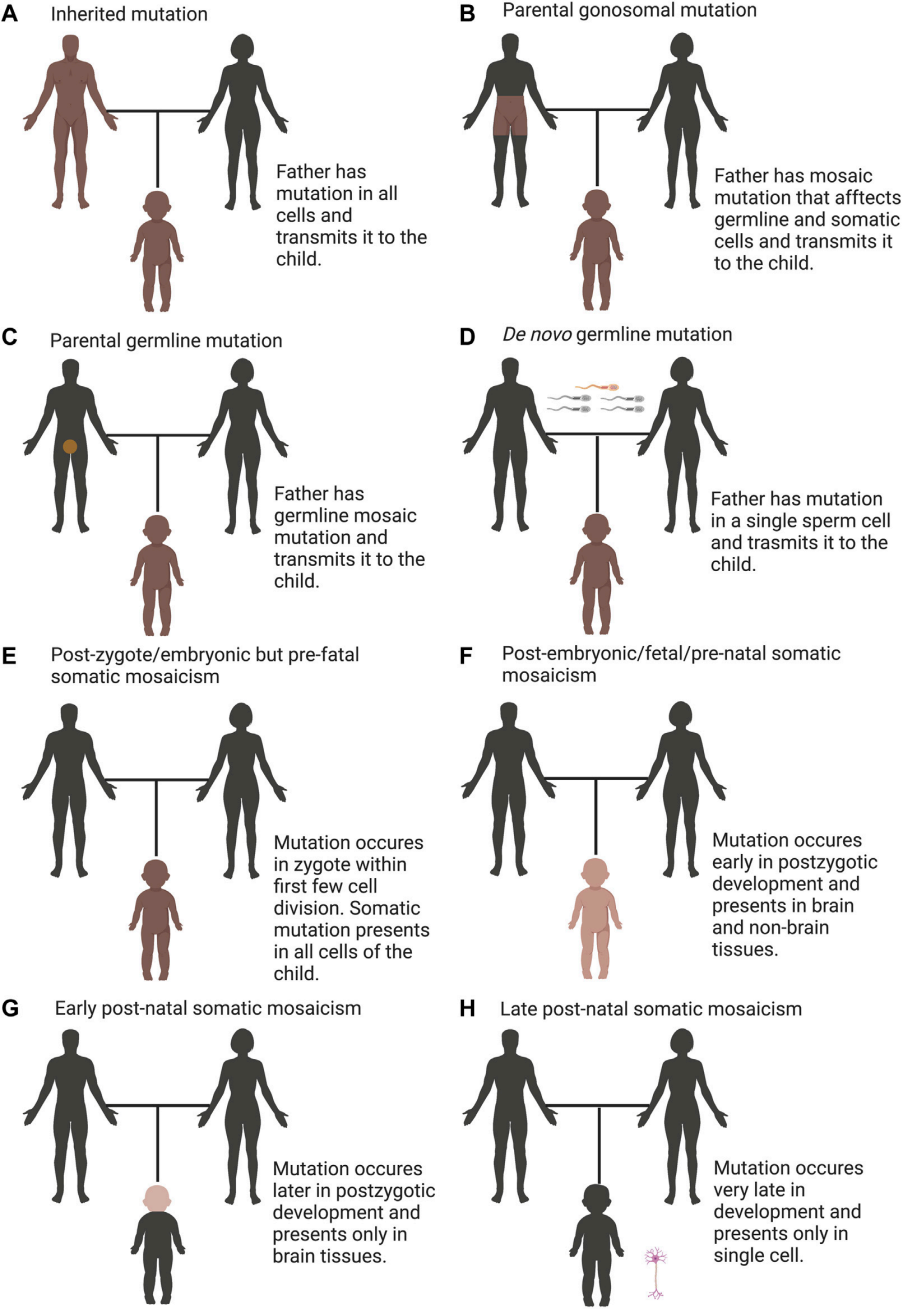
Overview of categories of mutations including inherited, *de novo*, and somatic variation. **(A)** Inherited mutations are constantly transmitted through the germline, which is detectable in all tissues of the child and their parent. **(B)** Parental gonosomal mutation is detectable in some tissues of the parents and in all tissues of the child, which is transmitted from a parent with mosaic mutation (combination of somatic and germline mosaicism). **(C)** Parental germline mosaicism is detectable in gametes of the parent and in all tissues of the child. **(D)** *De novo* germline mutation is detectable in all tissues of the child but not detectable in the parent. **(E)** post-zygote/embryonic but pre-fetal somatic mosaicism occurs in zygote within first few cell divisions such that the mutation presents in all cells that contribute to the embryo, is detectable in all tissues of the child but not detectable in the parent. **(F)** post-embryonic/fetal/pre-natal somatic mosaicism, which is present in nonbrain and brain tissues, occurs early in post-zygotic development, is detectable in some tissues of the child but not detectable in the parent. **(G)** early post-natal somatic mosaicism, which is present only in the brain, occurs later in post-zygotic development, is detectable only in brain tissue of the child but not detectable in the parent. **(H)** late post-natal somatic mosaicism, which occurs very late in post-zygotic development, is detectable only in single cell of the child, which requires single-cell sequencing to detect but not detectable in the parent. In all panels, brown denotes the mutation and darker shades designate increasing degree of mosaicism.

Genetic variations that cause neurological, neuropsychiatric and neurodevelopmental diseases are generally considered as either inherited or *de novo* germline mutations. Inherited mutations are present in one or both parents and in all tissues of the affected individual (Figure 1A). Hence, the mutation can be conveniently assayed in any tissue of the affected offspring. Despite the presence of inherited mutations in essentially all cells, they may affect some tissues more than others, depending upon when and where the gene involved exerts its crucial roles.

The mutation rate of single-nucleotide variants in humans to be approximately 1 × 10^−8^ mutations per generation, giving rise to 45–60 *de novo* mutations (DNMs) per genome (Roach et al., 2010; Kong et al., 2012; Michaelson et al., 2012; Campbell and Eichler, 2013). Mounting evidence shows the importance of DNMs mutations in neuropsychiatric and pediatric disorders (Awadalla et al., 2010; O’Roak et al., 2011; O’Roak et al., 2012; Devlin et al., 2012; Michaelson et al., 2012; O’ Roak et al., 2012; Sanders et al., 2012; Veltman and Brunner, 2012). DNMs are not detectable in either parent of an affected offspring. These mutations are typically existent in the sperm or egg of one parent and are not evident in blood taken of those parents, however, once transmitted to the embryo, the mutation can be detectable in any tissue of the affected individual (Figure 1D).

Any genetic variation in the genomes of germinal cells within an individual is termed as germline mosaicism. Germline mosaicism is a driving force behind evolution and a fundamental cause of genetic diseases. A human zygote receives 50% of its genome from the father through the sperm and the other 50% from the mother via the oocyte. In addition to the genomic material passed on from generation to generation, we are born with a little number of novel mutations—that arose either during the formation of the gametes or post-zygotically (Lynch, 2010; Roach et al., 2010). Each individual’s genome carries approximately one *de novo* germline mutation in their protein-coding region of the genome that are not evident in their parents’ somatic cells (Awadalla et al., 2010; O’Roak et al., 2011; Devlin et al., 2012; Sanders et al., 2012). Such mutations may manifest in individuals with neurodevelopmental and neuropsychiatric conditions such as autism spectrum disorder (ASD) (Awadalla et al., 2010; O’Roak et al., 2011; Devlin et al., 2012; Sanders et al., 2012). These *de novo* germline mutations are probably damaging, suggesting that many of these *de novo* germline mutations are associated with diseases. The contribution of *de novo* mutations to a disease will be enhanced if the mutations have a large negative effect on survival and reproduction (Veltman and Brunner, 2012). Neurodevelopmental or neuropsychiatric diseases, including autism—often arise by *de novo* mutations that are not evident in either of the parents.

*De novo* mutations can also occur post-zygotically during embryo development, through fetal development (beginning on the 9th week post-fertilization), and post-natally, through to death. These mutations lead to individuals who are mosaic, the presence of genetically distinct cells within a single organism (Youssoufian and Pyeritz, 2002; Biesecker and Spinner, 2013). These mutations are considered *de novo* because they are not evident in the parents or in the zygote of the affected individuals but are more precisely characterized as somatic mutations. Somatic mutations trigger antigenic variation, and have long been associated to cancer, with spans of studies and technological advances supporting and illuminating their role (Philp et al., 2001; Martincorena and Campbell, 2015). The latest progresses in DNA sequencing have shown mosaicism to be more prevalent than previously assumed in humans (Macosko and McCarroll, 2012; Biesecker and Spinner, 2013; Campbell et al., 2015; Forsberg et al., 2017), whether occurring in development or ageing, with the term ‘somatic evolutionary genomics’ used to explain the study of the accrual of somatic mutations in the body (Frank, 2010). Somatic mutations can indeed be used to reconstruct the developmental cell lineage in an organism (Frank, 2010; Woodworth et al., 2017; Coorens et al., 2021; Fasching et al., 2021; Park et al., 2021; Spencer Chapman et al., 2021). The proof of the involvement of somatic mutations in a wide range of neurodevelopmental and neuropsychiatric disorders is already robust (McConnell et al., 2017). A role of somatic mutations in neurodegenerative diseases has been constantly postulated (Pamphlett, 2004; Frank, 2010; Van Broeckhoven, 2010; Proukakis et al., 2013). Moreover, variable levels of mutations have been reported in somatic human tissues, including the brain, skin, and blood, and in patient-derived induced pluripotent stem cells (Baillie et al., 2011; Abyzov et al., 2012; Evrony et al., 2012; Macosko and McCarroll, 2012; O’Huallachain et al., 2012). It is well-known that germline mosaicism is difficult to evaluate, especially if a maternally originated mutation is suspected, because it is difficult to derive eggs from ovaries besides the very fact of it being a highly invasive procedure. Then, germline mosaicism is suspected when two or more affected children are born to apparently unaffected parents (Gajecka, 2016). When a mosaicism in the germ line was identified because of the birth of more than one affected child, the mutation was also present in somatic cells in half of the cases (Zlotogora, 1998).

## Types of mosaicism

Specific types of mosaicism describe which parts of the body harbor the variant cells and the potential for transmission to offspring. A new classification of genetic mosaicism depending on the distribution and pattern of the mosaicism, the affected tissue, the pathogenicity of the variant, the postzygotic mutational mechanism and the direction of the change (benign to pathogenic vs. pathogenic to benign) were reviewed elsewhere (Martínez-Glez et al., 2020). Apart from somatic mosaicism, which indicates to mutations that occur in somatic cells and are not evident in germ cells (Figures 1E–H), germline mosaicism (also known as gonadal mosaicism) (Figures 1C,D), which indicates to mutations that occur in germ cells and are not evident in somatic cells and gonosomal mosaicism (combination of somatic and germline mosaicism) where mutations evident in a subset of somatic and germ cells (Biesecker and Spinner, 2013; D’Gama and Walsh, 2018) (Figure 1B). Gonosomal mosaicism arises due to two possible mechanisms: the mutation arises in a germ cell that continues to divide. The other path to gonosomal mosacism is that the mutation arises very early in a somatic cell before the separation to germinal cells and hence evident in both germinal and somatic cells (Jansen et al., 1994; Zlotogora, 1998).

Although these categorizations are beneficial in an exceedingly practical sense, we concede that they cannot be decisively assigned due to the restrictions of tissue sampling. It is difficult or impossible to disentangle the differences. As an example, the detection of germline mosaic patient is usually based on the detection of a mutation in various germ cells (usually sperm) and on the lack of the mutation in skin fibroblasts and/or peripheral blood; however, the incidence of the mutation in other somatic cells cannot strictly be excluded.

Disease-associated somatic mutations can only be existent in the affected tissue but absent in other tissues, including the blood of the same patient (Rivière et al., 2012a; Lee et al., 2012; Poduri et al., 2012). The latter obscures the mutation from detection using routine diagnostic profiling. Considering the timing of mutation, somatic mosaicism can be classified as: i) post-zygote/embryonic but pre-fetal somatic mosaicism- where mutation arises within the zygote or during the primary mitotic divisions, such all cells that contribute to the embryo carry the mutation. This mutation is not detectable in the parent but can be identified in all tissues of the child (Figure 1E); ii) post-embryonic/fetal/pre-natal somatic mosaicism where mutation occurs early in development (following the blastula stage of 64 cells is the beginning of the fetal stage), may be existent in a high percentage of cells across multiple tissues (brain and non-brain tissues) and is not detectable in the parent but identified relatively easily in some tissues of the child (Figure 1F); iii) early post-natal somatic mosaicism- where mutation arises late in development, may be evident in a proportion of cells in only one tissue (brain) and can be identified only if tested, which is not detectable in the parent (Figure 1G); and iv) late post-natal somatic mosaicism- where mutation occurs in a post-mitotic cell like a neuron, is not evident in the parent and can be identified only in that neuron of the child, which requires ddPCR or single-cell sequencing (D’Gama and Walsh, 2018) (Figure 1H). Subsequent mosaicism can arise during infancy, childhood, teen, young adult, adult, and aged adult hood.

The discrepancy between germline and somatic mosaicism is based on the finding of genetically different populations of cells in the germ-line and somatic tissues, respectively (Hall, 1988; Youssoufian, 1996). Mosaicism that arises during embryogenesis can be coexist as both germ-line and somatic mosaicism in the same individual. Coexistence depends on the specific cell affected and the timing of the development of the mosaicism-inducing event. Reckoning on various factors, like the gene involved, the location in the gene (Monroe et al., 2022; Zhang, 2022) and/or the degree and tissue location of mosaicism, the carrier of a somatic and germline mosaicism may be asymptomatic or may evident with various symptoms of the disease. In most cases, symptoms evident in the mosaicism carrier were milder than that present in the offspring (Zlotogora, 1998).

In germ cells, *de novo* mutations will almost always present as mosaicism if it occurs before meiosis, and, depending on the timing, may be evident in a very few to up to almost half of the germ cells. Human families are usually small. So, it is plausible that, in most of the cases in which a mutation is transmitted to more than one child, the mutation is evident in a significant number of germ cells and therefore must have occurred very early after a few divisions of the committed germ cells (Zlotogora, 1998). Whereas mutations during post-fertilization cell division and throughout life can contribute to somatic mutations, transmitted instability must involve germline mutations.

It has been anticipated that *de novo* germline mutations derive from errors in DNA replication during gametogenesis, specifically in sperm cells and their precursors. However, *de novo* mutations during spermatogenesis can also arise due to ineffective repair of spontaneous DNA lesions, as constant proliferation and short periods between cell divisions can translate into there being less time to repair these lesions (Goriely and Wilkie, 2012; Gao et al., 2016). Likewise, in oogenesis, ineffective repair mechanisms coupled to spontaneous DNA mutations might play a prominent role (Gao et al., 2016). Therefore, while the *de novo* mutation rate could be a reflection of the replication error rate and also the number of mitoses a cell has experienced, this number is additionally influenced by the quantity of time between mitoses and therefore the proficiency of the DNA repair (Gao et al., 2016).

## The origin of mosaicism

The origination of genetically distinct cells from a single zygote necessitates *de novo* post-zygotic mutational events (Veltman and Brunner, 2012) as the cause of mosaicism, and these mutations can result in sporadic disease. *De novo* mutational events can also arise pre-zygotically; where mosaic parent, usually unaffected, but the mutation might be inherited in the zygote and possibly in all cells of the developing offspring also to result in the creation of a *de novo* disease phenotype (Veltman and Brunner, 2012).

Mosaicism can also arise from either meiotic or mitotic circumstances. If a meiotic error has occurred and revised at the cleavage stage, it will cause mosaicism. The meiotic errors are usually more damaging because of the initial onset of the abnormality, which permits the mosaicism to dominate. Nevertheless, mitotic errors may be as extreme as meiotic errors depending on their time of occurrence. Mosaicism could be present through all embryonic tissues if a mitotic error occurs in the first one or two divisions. However, if the mitotic errors occur further along in development, the mosaicism could be tissue specific.

Genetic variation between individuals can occur due to chromosome segregation or DNA replication errors, leading to CNVs, chromosome aneuploidy, genomic rearrangements, repeat expansions, single-nucleotide variation, and microsatellite instabilities. These mutational processes can happen at any stage of development; in differentiating cells, stem cells, and in terminally differentiated somatic cells, leading to mosaicism (Forsberg et al., 2017), and have now been reported in normal brain (McConnell et al., 2017). DNA sequence characteristics (e.g., CpG dinucleotides) and genomic architectural features (e.g., inverted and direct repeats) can accelerate genome instability and susceptibility to mutation. Mosaicism can also occur during either repair of DNA damage (single-strand breaks (SSBs) and double-strand breaks (DSBs), or DNA replication (Lindahl and Wood, 1999; Hoeijmakers, 2001; McKinnon, 2009; Rulten and Caldecott, 2013). DNA damage may cause transient chemical lesions, but inaccurate repair of these damages cause somatic mutation (Vijg and Suh, 2013). Furthermore, DNA damages caused by exogenous sources, such as tobacco smoke and other carcinogens, may cause somatic mosaicism in targeted tissues. Accumulative exposures to exogenous mutagens, together with ongoing development of the organism, tissue regeneration, and cell proliferation and renewal, caused accumulation of mutations with age. Moreover, previous study proposed that nonallelic homologous recombination–predicted inversions, such as structural variations, are mosaic and seem to accumulate as the individual ages (Flores et al., 2007). L1 transposition during embryogenesis can caused mosaicism (Kano et al., 2009). Several unbalanced translocations seem to originate post-zygotically, apparently appearing *de novo* during embryogenesis in a manner that is based on homologous interspersed transposable elements as substrates (Robberecht et al., 2013), and recombination-restarted replication forks contribute to other post-zygotic mutational events (Mizuno et al., 2013).

## Cellular and DNA metabolism through human gametogenesis

**FIGURE 2:**
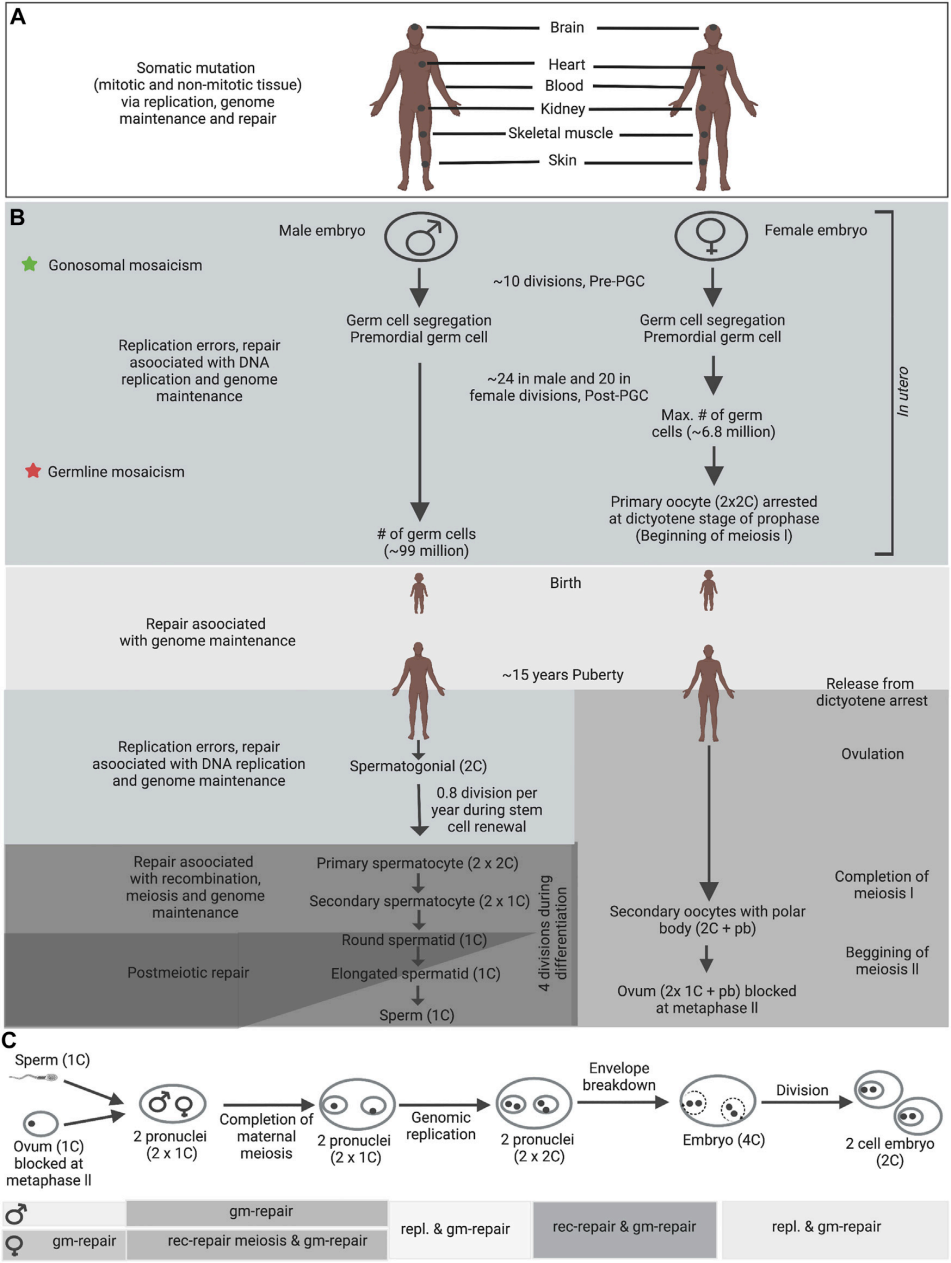
Germline and somatic DNA metabolic processes in human. **(A)** Somatic mosaicism in patients with a genetic disease can exhibit both pre-natal and post-natal tissue specific mosaicism. **(B)** Depending on the timing of mutations during embryonic development, different types of germline mosaicism can arise; star signs indicate different stages at which mutations can arise and the consequential types of mosaicism. Germline mosaic variants, which were apparent within the parents’ blood, were possibly established before mesoderm tissue separation from PGCs within the parents (green stars). One potential explanation for mosaic mutations that are only shared by siblings—were not apparent in the parents’ blood—is that the mutations arose after separation of PGCs from mesoderm in the mosaic parents (red stars). **(C)** Fertilization and also the steps leading to the two-cell embryo, which includes the development of two pronuclei, the completion of maternal meiosis II, decondensation of the paternal genome, DNA repair, gonomeric DNA replication within two haploid pronuclei, the breakdown of pronuclear envelopes, syngamy and cleavage (Gianaroli, 2000). Gonomeric duplication is that the only haploid DNA replication within the diploid metazoan life cycle. DNA metabolic processes appearing during each developmental stage are denoted by graded shading. Abbreviations: rec-repair - recombination-associated repair; gm-repair - genome maintenance repair; repl. - replication errors and replication associated repair; pb, polar body. Information compiled from previously published studies (Drost and Lee, 1995; Gianaroli, 2000; Zenzes, 2000; Baarends et al., 2001). These processes can differ prominently between different species. Figure adapted from Pearson (2003).

While mutations during post-fertilization cell divisions and throughout life can contribute to somatic instability (Figure 2A), transmitted instability must encompass germline mutations (Figure 2B). Numerous genetic diseases are inherited primarily through either maternal or paternal transmissions. Each parent-of-origin mutation bias is driven by complex processes specific to sperm or oocyte development. Mosaic mutation could occur in proliferating, arrested meiotic cells or within the relatively dormant haploid germ cells (Figure 2). Knowledge about the timing of mutation events during germ-cell development in patients is critical to understanding particular important mutation and its transmission in affected families. It will also suggest which DNA metabolic process facilitates instability. During gametogenesis, germline mosaicism might arise due to highly specialized DNA metabolic activities that engage stage-specific expression of repair, replication or recombination factors, replication programs, expression profiles and epigenetic modifications. Previous studies demonstrated that many repair, replication or recombination genes show reformed or particular expression during gametogenesis (Drost and Lee, 1995; Kamel et al., 1997; Richardson et al., 2000; Baarends et al., 2001; Velasco-Miguel et al., 2003). Replication mediated mutation can be complex and could vary between different tissues and/or developmental stages. Mutation events must appear at some point between segregation of the primordial germ cells, development to puberty, or during the life-long post-pubertal spermatogonial stem-cell divisions (Figure 2). The large number of life-long spermatogonial cell divisions might push the paternal mutation bias. As mutations are evident in human pre-meiotic diploid cells, the early mutation event must involve DNA replication fork errors, genome maintenance repair or replication-associated repair. Additional mutations might involve post-meiotic genome maintenance or recombination events (McKinnon, 2009; Rulten and Caldecott, 2013; Vijg and Suh, 2013).

The genome of the haploid spermatids is re-packaged with protamine at the completion of spermatogonial proliferation and meiosis, thereby entering a comparatively inactive or ‘sleeping’ state, and ultimately becoming spermatozoa. After meiosis, an additional phase of expansion might happen and probably through to elongating spermatids. Spermatogonia and primordial germ cells undertake genome replication and are subject to DNA replication errors, genome maintenance repair, and repair related to replication (Figure 2). The mitotically inactive post-natal pre-pubertal stages undergo genome maintenance repair. Later stages are subject to genome maintenance repair, meiotic recombination and, ultimately, chromatin decondensation (coincident with dormancy) (Figure 2C).

Although the timing has not been thoroughly established, it is assumed that both male and female germ-cell segregation is occur between day 5–12 post-fertilization. Primordial germ cells (PGCs) emerge from the epiblast, which requires ∼10 cell divisions after fertilization (Campbell et al., 2014b). Notably, initial few cell divisions of embryogenesis is relatively mutagenic, leading to germline and somatic mosaic mutations in mice (Lindsay et al., 2019) and humans (Huang et al., 2014; Acuna-Hidalgo et al., 2015; Rahbari et al., 2016a; Ju et al., 2017; Lim et al., 2017), as well as to mutations that are discordant between monozygotic twins (Dal et al., 2014; Jónsson et al., 2017). During first few cell divisions, male and female embryos seem to have similar mutation rate (according to models estimating the mutation rate during gametogenesis, approximately 0.2–0.6 mutations per haploid genome per cell division) (Rahbari et al., 2016a). After their specification, PGCs expand to form the complete population of primary oocytes and the pool of spermatogonial stem cells in female and male embryos, respectively (Campbell et al., 2014b; Rahbari et al., 2016a). According to computational modeling, the mutation rate during the expansion of PGCs to oogonia or spermatogonia is comparable in both sexes, with approximately 0.5–0.7 mutations per haploid genome per cell division (Rahbari et al., 2016a). However, spermatogonial stem cells undergo post-pubertal spermatogonial cell divisions throughout life (23 cell division per year during stem cell renewal), maintaining the spermatogonial stem cell pool while generating differentiated spermatogonial cells which produce sperm cells through an extra round of mitosis followed by meiosis (Crow, 2000). By disparity, oogenic meiosis initiates *in utero* and is halted in dictyotene for years, re-starting only minutes before ovulation, after which meiosis I is complete. Meiosis II then starts after the completion of meiosis I and is not completed until fertilization. Thus, oocytes undertake only one extra round of DNA replication in their evolution, after the completion of PGC expansion in embryogenesis, to a mature ovum. On the other hand, spermatogonial cells can experience hundreds of rounds of cell division and DNA replication before their maturation to sperm cells. More than 75% of all germline *de novo* point mutations appear on the paternal allele (Francioli et al., 2015; Rahbari et al., 2016a). Both at the population level and within the same family, advanced paternal age at conception has been established as the major factor that linked to the increase in the number of *de novo* mutations in the offspring (Kong et al., 2012; Rahbari et al., 2016a; Goldmann et al., 2016). Male germ cells continue to divide throughout life, which allows the gradual accumulation of mutations due to DNA replication errors and repair defect of non-replicative DNA damage between cell divisions (Gao et al., 2016). Moreover, the proficiency of endogenous defense systems against radical oxygen species and of DNA repair mechanisms might also weakening with age (Paul and Robaire, 2013; Goriely, 2016). In contrast to post-meiotic male germ cells, DNA recombination and repair activities are linked with arrested and/or resting oocytes (Baarends et al., 2001; Nouspikel and Hanawalt, 2002).

**FIGURE 3:**
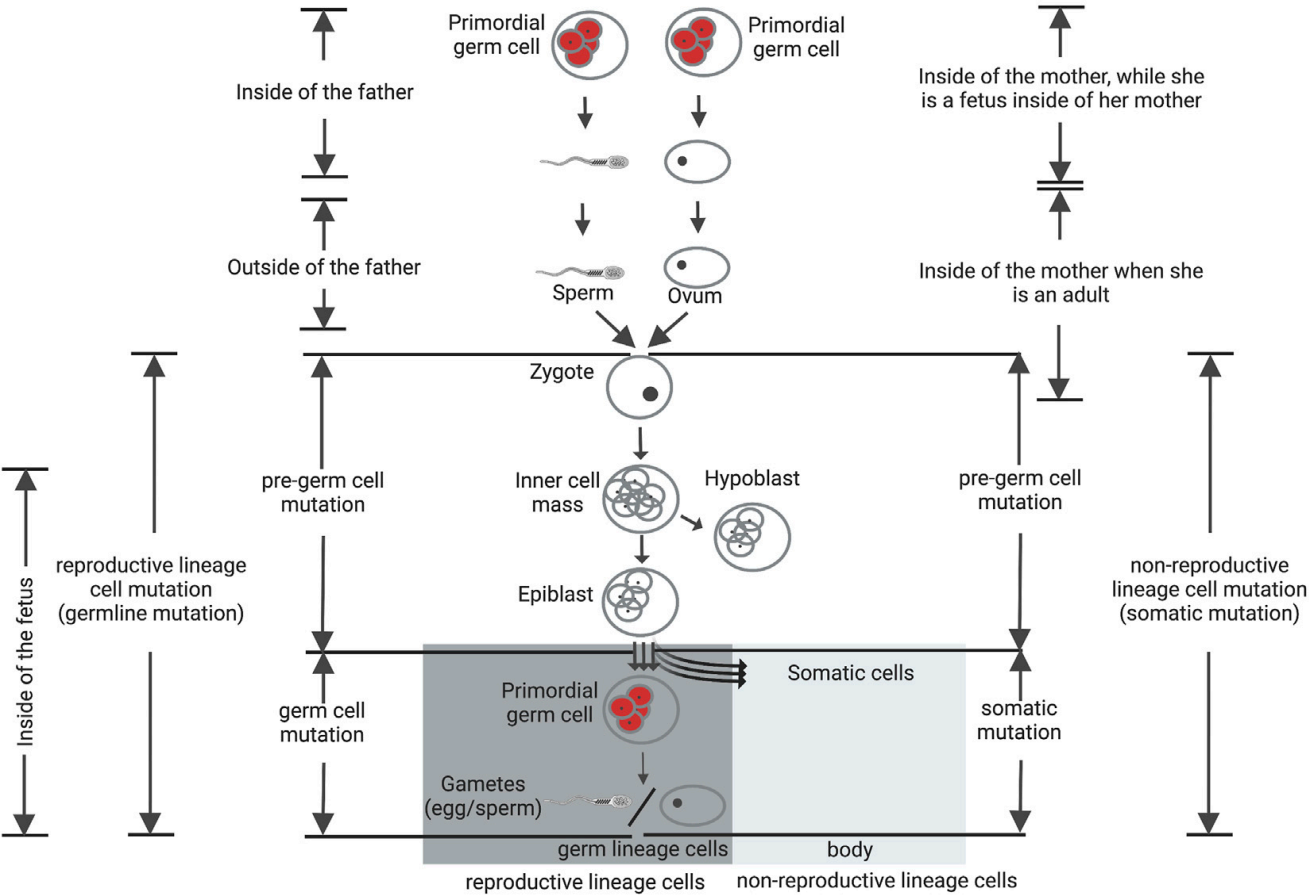
Reproductive and non-reproductive lineage cells and *de novo* mutations. Mutations that occur in the reproductive lineage from a fertilized egg to gametes can be defined as reproductive lineage cell mutations and categorized into two groups: pre-germ-cell-stage mutations (gonosomal mutation) and germ-cell-stage mutations. The germ-cell-stage mutations arise in the primordial germ cells (pGCs) and their offspring after the point of divergence from somatic cells, usually being designated as germline mutations. Gonosomal mutations arise before this point of divergence. These mutations can exist in both somatic cells and gametes at the same time. The mutations that arise in the non-reproductive lineage cells, after the stage of divergence from germ lineage cells, are designated as somatic cell mutations and are unable to transmit to the offspring. Figure adapted from Sakumi (2019).

Germ cell specification process occurs in mammals over the time of gastrulation. Being separated from nonreproductive cell lineage cells, the reproductive lineage cells are able to form germ lineage cells. The reproductive lineage originates from a fertilized egg located in the mother’s body. For the preparation of the future body, cell propagation continues and an epiblast is created (De Felici, 2013; Behringer et al., 2014). Following invagination of the epiblast, several originated cells find a place in the wall of the yolk sac and successively migrate as primordial germ cells (PGCs) to the gonadal ridge. Somatic lineages are also differentiated around the same time, and also have their origins within the epiblast (Figure 3, adapted from Sakumi (2019). Non-reproductive lineage cell mutations, that arise after the stage of divergence from germ lineage cells, will be restricted to that individual. These are designated as somatic cell mutations and are unable to transmit to the offspring. On the other hand, mutations that occur in the reproductive lineage from a fertilized egg to gametes can be defined as reproductive lineage cell mutations. Such mutations can transmit to the next generation, but do not exist in the parents, are often called germline (*de novo*) mutations.

Reproductive lineage cell mutations are classified into two groups: pre-germ cell-stage mutations and germ-cell-stage mutations (Sakumi, 2019). The latter group arise during or after PGCs specification, may be absent from somatic cells but present in multiple resulting gametes (Biesecker and Spinner, 2013; Campbell et al., 2014b, 2015; Acuna-Hidalgo et al., 2015; Rahbari et al., 2016b; Tang et al., 2016; Jónsson et al., 2018). These variants can consequently be existing in more than one offspring as evident *de novo* mutations. In contrast, the former group of mutations arise before PGCs specification, but likely following zygote fertilization. Mutations that arise prior to PGCs specification can be exist in both somatic cells and gametes at the same time, characterize as “gonosomal” variants that possibly aroused early during the early post-zygotic development of second-generation individuals (Campbell et al., 2014b, 2014a, 2015; Besenbacher et al., 2015; Rahbari et al., 2016b; Jónsson et al., 2018). Overall, gonosomal mutations, that aroused during the early cell divisions of the offspring, rather than in a single parental gamete, consist of approximately 10% of candidate *de novo* germline mutations (Sasani et al., 2019). Therefore, to define the biological significance of *de novo* mutations in sexual reproduction of higher multicellular organisms such as mammals, it is essential to define when and where these mutations originated.

## Selfish spermatogonial selection

The fitness of the spermatogonia themselves can be affected by spermatogonial mutations. Some mutations in critical genes might lead to the eradication of the spermatogonial lineage, while other mutations can result in selective advantages and lead to clonal expansion, the outgrowth of the specific cell lineage. The later behaves selfishly and are therefore denoted as “selfish mutations” (Goriely et al., 2009). Due to the expansion of mutated spermatogonia that happens in the testes of healthy men during aging (Meng et al., 2000; Goriely et al., 2009; Giannoulatou et al., 2017), the quantity of mutated sperm raises continuously at higher rates than genome-wide paternal age effect. If these mutations are passed on to offspring, most of the cases they will trigger developmental disorders. The frequency of these developmental disorders shows an exponential increase with paternal age at conception (Goriely et al., 2009). This is much higher than observed increase for other *de novo* mutations related disorders (Arnheim and Calabrese, 2009).

## Timing of *de novo* mutations

During development through aging, mutation timing could lead to mosaicism levels fluctuating from less than heterozygous to approaching homozygous level (Mohiuddin et al., 2022). The distribution of somatic mutation in the whole body is regulated by the developmental timing of mutations. Mutation loads between tissues could uncover timing of mutation and may shed light on disease etiology and clinical variability.

Mutation loads greater than heterozygosity (approaching homozygosity) support second-hit mutation theory, which produce mosaicism (Poduri et al., 2013). Moreover, varying levels of tissue specific mutation burden were evident between the post-mortem tissues, both above and below heterozygosity (Mohiuddin et al., 2022). Individuals with less than heterozygous mutations in at least one tissue argue against mutations having arisen in the transmitting germline. Moreover, the highly variable mutation loads between tissues, that can approach homozygous level, suggest extremely high rates of post-zygotic and probably post-natal somatic mutations.

**FIGURE 4:**
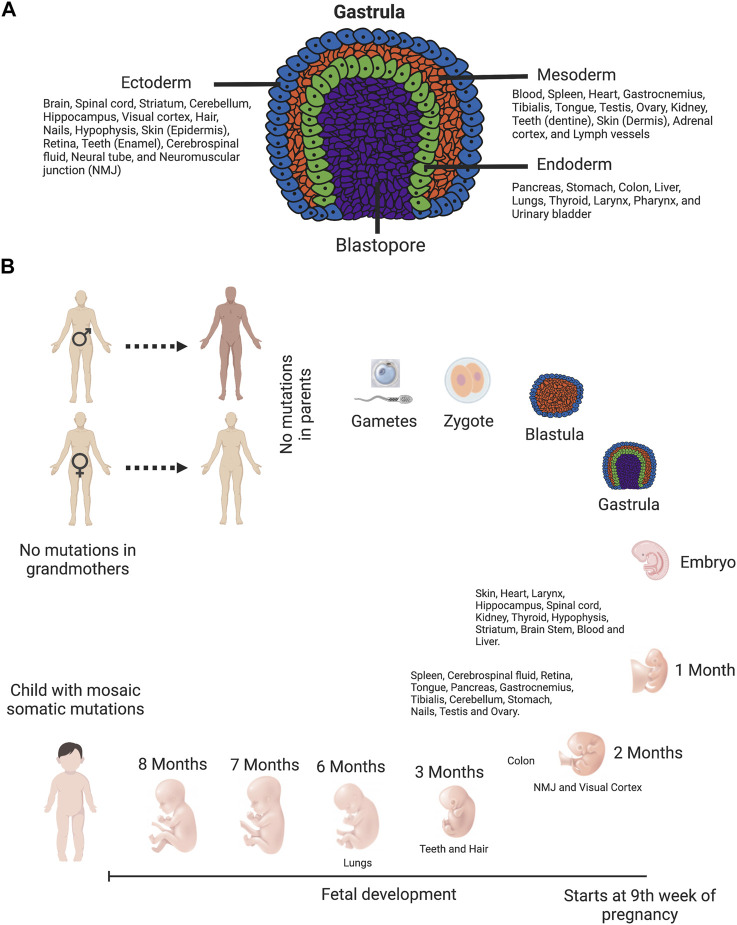
Timing of *de novo* mutations: they can happen anytime. **(A)** Diagram of gastrula—the embryo with three primary germ layers (ectoderm, mesoderm, and endoderm). This diagram is color-coded: ectoderm, blue; mesoderm, orange; endoderm, green; and blastopore, purple. Cells in ectoderm, mesoderm and endoderm differentiate into tissues and embryonic organs. The ectoderm contributes to the nervous system and the epidermis, among other tissues. The mesoderm contributes to the muscle cells and connective tissue in the body. The endoderm contributes to the gut and many internal organs (Pansky, 1982). **(B)** Human timeline of development features and approximate developmental timing of various tissues. Tissues are arranged depending on the approximate time of development. The single-cell zygote, which proceeds through cleavage divisions and the morula stage to form the blastocyst, is arose by the fertilization of a mature oocyte by a sperm cell. The embryo is originated from the inner cell mass of the blastocyst. During the process of gastrulation, these cells differentiate to form the three germ layers (ectoderm, endoderm, and mesoderm), which differentiate into tissues and embryonic organs. Following birth and sexual maturation, mature sperm and oocytes are produced by the completion of meiosis in adult animals. Schematics prepared using bio-render software.

The single-cell zygote, which proceeds through cleavage divisions and the morula stage to form the blastocyst, is arose by the fertilization of a mature oocyte by a sperm cell. The embryo is originated from the inner cell mass of the blastocyst. During the process of gastrulation, these cells differentiate to form the three germ layers (ectoderm, endoderm, and mesoderm), which differentiate into tissues and embryonic organs. The ectoderm contributes to the nervous system and the epidermis, among other tissues, whereas the mesoderm contributes to the muscle cells and connective tissue in the body, and the endoderm contributes to the gut and many internal organs (Figure 4A) (Pansky, 1982). Depending on the timing at which a *de novo* mutation occurs during embryonic development (Figure 4B), it could be organ specific or be evident at different levels in numerous tissues (Youssoufian and Pyeritz, 2002).

## Parental influence on the rate and spectrum of human *de novo* germline mutations

The higher rate of mutation for the hemophilia-associated gene in men than in women was noted in 1947 by HALDANE (1947). Haldane’s observation was decades later confirmed by whole genome sequencing that the male germ line is more mutagenic (Roach et al., 2010; Conrad et al., 2011; Kong et al., 2012; Michaelson et al., 2012; Venn et al., 2014). Mounting evidence have established the observation of the paternal age effect in humans (Francioli et al., 2015; Rahbari et al., 2016a; Goldmann et al., 2016, 2018; Wong et al., 2016; Jónsson et al., 2017). Each additional year in father’s age at conception, on average, results in ∼2 additional DNMs in the child (Kong et al., 2012). Consistently, the chance of dominant genetic disorders in the child rises with increasing paternal age (Goriely and Wilkie, 2012; Momand et al., 2013). The increasing number of cell divisions in the male germ line might be the cause of the paternal age effect (Penrose, 1955; Crow, 2000; Kong et al., 2012). To confirm the continuous generation of sperm cells, the male germline undergoes very frequent genome replications. It has been predicted that in a 20-year-old male the male germ line has experienced 153 genome replications (10 very early embryonic divisions, plus 24 early embryonic divisions, plus 5 years of sperm production each with 23 divisions (Heller and Clermont, 1963)- assuming a sperm production onset at the age of 15 years, and finally 4 replications for spermatogenesis) with the number mounting to 613 genome replications in a 40-year-old male. The replication frequency increased by a factor of ∼four between the parental ages of 20 and 40 years. However, these estimated numbers of cell divisions are not proportionate with the observations on DNM accrual (Goldmann et al., 2019). Several studies have reported this unsolved mismatch correlation between genome replications and the number of DNMs (Ségurel et al., 2014; Forster et al., 2015; Gao et al., 2016, 2019; Scally, 2016).

Considering recent observations of DNM accumulation and single-cell transcriptome analyses of the spermatogenic epithelium, Goldmann et al. (2019) suggest that estimated rate of spermatogonium replication may have been constructed on wrong assumptions. In the genome-replication model, the estimate of 23 divisions per year is based on the observations by in the 1960s Heller and Clermont (1963). They showed that one division cycle lasts 16 days, which would conclude to 23 divisions per year, assuming that there are no interruptions or resting phases between the divisions. However, it has recently been observed by several group that the nature of spermatogonial self-renewal is driven by stochastic cell transitions, including cell stages without replicative activity (Hara et al., 2014; Krieger and Simons, 2015; Sharma et al., 2019). Recent studies on single cell transcriptome supported such nonhierarchical model of spermatogonial self-renewal with stochastic, continuous oscillation between cell stages (Guo et al., 2017, 2018; Green et al., 2018; Hermann et al., 2018; Wang et al., 2018). A spermatogonium division model has been proposed and its parameters have been estimated by a fit to DNM accumulation data (Scally, 2016). According to this newly proposed model, the yearly increase of spermatogonium divisions would be only 0.8 instead of 23 divisions per year. Consequently, the number of germline divisions for sperm of 20-year-olds and 60-year-olds would be 42 and 74, respectively. This represents an increase by a factor of 1.76, which would be closer to the increase in DNM numbers. However, recent study has reported a speculative sign of a maternal age effect on the quantity of mutations on the paternal genome, which in favor of the hypothesis that mother age at conception effects mutagenesis in early embryonic development of her child (Gao et al., 2019).

The number of DNMs in offspring rises with both paternal and maternal age, and that some genome regions show enhancement for maternally derived DNMs (Goldmann et al., 2016). The number of DNMs from mothers rises by 0.37 per year of age, which is one-fourth of the 1.51 per year from fathers (Francioli et al., 2015; Rahbari et al., 2016a; Jónsson et al., 2017). It has been reported that this ratio of paternal to maternal is independent of the age of the parents (Gao et al., 2019), as maternal DNMs also accumulate during aging, although the rate of accumulation is lesser than for the paternal allele. Several recent studies have confirmed the maternal age effect on DNMs (Goldmann et al., 2016; Wong et al., 2016; Jónsson et al., 2017; Gao et al., 2019). Due to the production of oocytes ceases prenatally, and no more genome replications occur, the mechanisms that trigger maternal aging-associated DNMs must fundamentally differ from those underlying the paternal aging-associated DNMs. On this foundation, recently detected maternal age effect (Goldmann et al., 2016; Wong et al., 2016; Jónsson et al., 2017; Sasani et al., 2019) has been interpreted as reflecting the accrual of DNA lesions or damage induced mutations in (primary) oocytes during the lengthy meiotic arrest phase (Ségurel et al., 2014; Gao et al., 2016; Goriely, 2016; Jónsson et al., 2017). However, other explanations for a maternal age effect cannot be excluded (Wong et al., 2016). For example, it could be an effect on post-zygotic mutations (Wong et al., 2016). Recently a positive association between the number of DNMs on paternal chromosomes and maternal age was reported which supports the hypothesis that the age of a mother at conception affects the post-zygotic mutation rate in the developing embryo (Gao et al., 2019).

Recent evidence suggest that mutations can accumulate at a high rate without cell division (Lodato et al., 2018; Abascal et al., 2021). By characterizing the mutational landscape of post-mitotic neurons and polyclonal smooth muscle, Abascal et al., confirm that neurons accumulate somatic mutations at a constant rate throughout life without cell division, with similar rates to mitotically active tissues (Abascal et al., 2021).

## Mutational signatures underlying specific mutational processes

A pattern of mutations that is specific to a mutational process occurring in a cell, tissue, or organism is defined as a “mutational signature” (Alexandrov et al., 2013). Mutations arising from different processes, such as DNA replication errors, failure to repair DNA damage, or exposure to mutagens, showed distinct mutational patterns. The various underlying biology of female and male gametogenesis ends up in differences in mutational signatures between maternally and paternally transmitted DNMs, respectively. Nucleotide type (Alexandrov et al., 2013; Aggarwala and Voight, 2016), sequence context (Alexandrov et al., 2013; Aggarwala and Voight, 2016), replication timing (Koren et al., 2012), functional constraints (Gudbjartsson et al., 2015; Aggarwala and Voight, 2016), apolipoprotein B messenger RNA-editing enzyme catalytic (APOBEC) polypeptide activity (Chan and Gordenin, 2015), and epigenetics (Polak et al., 2015; Rahbari et al., 2016a) have also been reported to affect the mutational landscape. The parent-of-origin specific mutation signatures that become more prominent with increased parental age, indicating to different mutational mechanisms in oogenesis and spermatogenesis (Goldmann et al., 2016). Consistent with biological difference, the mutational spectra of paternal and maternal DNMs are also different. The mutational spectrum, as opposed to the mutational frequency, was affected more by maternal than by paternal age (Jónsson et al., 2017). Paternal DNMs are enriched for C>T mutations and maternal DNMs are enriched for C>G mutations at CpG sites (Jónsson et al., 2017; Goldmann et al., 2018).The natures of *de novo* mutation from mothers change substantially with age, with a 0.33% increase in C>G *de novo* mutations per year. Surprisingly, these age-related changes are not evenly distributed through the genome (Jónsson et al., 2017). Some genome regions on chromosome 8, 9, and 16 are highly enriched for maternal DNMs (up to eight times compared with paternal DNMs) (Goldmann et al., 2016, 2018; Jónsson et al., 2017). The relative frequency of C>G mutations in these regions is drastically larger than among paternal DNMs, and even larger than in the remaining maternal DNMs. In contrast to female germline CpG sites, male germline CpG sites in cells are more favorably methylated, making the sites more prone to C>T transitions and spontaneous deamination (Kobayashi et al., 2013; Gao et al., 2019).

## Mutational clusters and hotspots

DNMs arise throughout the human genome, but intermittently several mutations can appear at a nearer distance than usual by casual distribution (Michaelson et al., 2012). Mutational clusters, the occurrence of DNMs in an individual on the same DNA molecule at a closer distance than usual, with numerous DNMs within short reciprocal distances, usually several kilobases ranging from 10 to 100 kb (Michaelson et al., 2012; Chan and Gordenin, 2015; Francioli et al., 2015). These mutations characterize a distinctive subset of 2–3% of DNMs rising due to a single mutational event, as the frequency of clusters is too high to be justified by the incidental co-occurrence of numerous independent sequential DNMs (Schrider et al., 2011; Michaelson et al., 2012; Terekhanova et al., 2013; Francioli et al., 2015; Besenbacher et al., 2016; Goldmann et al., 2016; Yuen et al., 2016).

Clustered DNMs are also unique in terms of their parental age effects and parent-of-origin characteristics. Clustered DNMs are equally rich on maternal and paternal alleles, which is very unlikely to other DNMs. In addition, the quantity of these mutations are strongly associated with maternal than with paternal age, increases quicker with the mother’s age than with the father’s (Jónsson et al., 2017; Goldmann et al., 2018). Moreover, mutational clusters display a distinctive mutational spectrum with a significant enhancement of C>G mutations at non-CpG sites (Francioli et al., 2015; Besenbacher et al., 2016; Goldmann et al., 2016, 2018), which is also significantly different between the parental alleles, with the distances between the DNMs of a maternal cluster are greater than for paternal clusters, and the maternal allele being more inclined to C>G mutations than the paternal. In the perspective of cancer, where it is known as “kataegis”, it has been proposed that clusters comprising C > G transversions could be linked to the formation of single stranded DNA in diverse cellular processes, such as dysfunctional replication forks and double-strand breaks (DSBs) (Sakofsky et al., 2014). Chromosomal rearrangements could also be another origin for some of these clusters. Previous studies show that the mutation rate for SNVs is prominent and SNVs can cluster in vicinity to the breakpoints of *de novo* CNVs (Neumann et al., 2010; Carvalho et al., 2013). Previous study on yeast supports the finding that DSB-induced replication is a source of mutation clusters (Sakofsky et al., 2014).

Unlike mutation clusters, that occur within one individual, mutational hotspots are considered overlapping loci that are mutated more frequently than usual in the population. Mutation hotspots in coding sequence has identified by recent research based on whole genome sequencing (WGS) datasets and modeling (Michaelson et al., 2012). The presence of mutational hotspots has also been established in a larger study that exhibited specific bins of 1 Mb within the human genome with elevated mutation rates (Goldmann et al., 2016).

## Male germline mosaicism to predict the recurrence risk of genetic diseases

Recently, Breuss et al. (2020), (2021) sequenced fathers’ sperm DNA to define paternal germline mosaicism that could improve risk predictions for families with children with autism. Prezygotic *de novo* mutation in an unaffected parent may exist in a mosaic fashion. Mosaic *de novo* mutation might be inherited in the zygote and possibly in all cells of the developing offspring, which may cause a *de novo* disease phenotype. Because *de novo* prezygotic mutations arise before fertilization in the parental germline, their existence is closely connected to the biology of germ cells. A DNM has been assumed to arise in a single sperm or egg during meiosis, and in such cases, the hypothetical possibility of recurrence of this mutation would be remarkably low in the subsequent children. However, the probability of germline mosaicism uplifts risk of recurrence of the same mutation in families higher than in the general population. The developmental timing and cell lineage affected, merged with the phenotypic consequences of the mutation, eventually regulate the tissue distribution of mosaicism and also the patterns of disease reoccurrence within families.

Breuss et al. (2020), (2021) measure the allele frequency of *de novo* single nucleotide variants (dSNVs) in sperm relative to that in blood and discovered that a large majority of the mutations are evident in sperm only or are enriched in sperm comparative to their abundance in blood. Depending on their findings, they conclude that germline mosaicism in fathers can be identified better by sequencing of sperm than does sequencing of blood (Breuss et al., 2020; Morrow, 2020). Only somatic cells are routinely assessed in genetic analyses, and a clue about possible germline mosaicism will be obtained if somatic mosaicism is detected, or if more affected siblings are born with an analogous *de novo* variant. The incidence of parental germline mosaicism has been reported in many disorders now. Recently, in case of routine genetic screening for germline mutation in fathers of patients with apparently *de novo* mutations, it has been suggested that analysis of sperm could more often be performed (Pacot and Pasmant, 2019).

The identification of a *de novo* mutation as the cause of disease in a patient has numerous implications for the patient and his family. In terms of family planning, the identification of a *de novo* mutation as the cause of disease in a child can be positive news with respect to recurrence risk. There is a low risk of recurrence of the mutation in an additional child for the parents with a post-zygotic mutation, anticipated as being the same as the population risk. Breuss et al. (2020) proposed a genetic testing whereby paternal sperm can directly be assessed for pathogenic variants which is previously transmitted and these variants may thereby be categorized as low risk for recurrence or high risk for recurrence. The authors further propose that in case of family planning, men may have their sperm DNA sequenced as a method of discerning high-risk mosaic mutations. Undoubtedly, this process may additionally be appropriate to sperm donor clinics.

## Somatic repeat instability

Expansion of gene-specific tandem repeat DNAs, like (CAG)n, (CGG)n, and (GGGGCC)n underlies over fifty neurological, neuromuscular and neurodegenerative diseases, including Huntington disease (HD), myotonic dystrophy (DM1), spinocerebellar ataxias (SCAs), fragile X syndrome (FXS), schizophrenia, amyotrophic lateral sclerosis (ALS) and autism (ASD) (Castel et al., 2010; DeJesus-Hernandez et al., 2011; Song et al., 2018; Course et al., 2020; Trost et al., 2020). The key mechanisms by which expansion of repeat DNA causes disease are R-loop formation followed by the activation of the DNA damage response and repeat- induced transcriptional gene silencing, repeat associated RNA translation of toxic repeat peptides, and RNA- facilitated gain of function through gelation and sequestration of RNA- binding proteins, gain of function of canonically translated repeat-harboring proteins. The severity of these mechanisms is amplified by somatic repeat instability, a process that differentially regulates repeat length across the cells and tissues of individuals over their lifespan (La Spada and Taylor, 2010; Nussbacher et al., 2019; Khristich and Mirkin, 2020), which impacts both disease age of onset and tissue specificity of pathogenic features. Several studies on somatic instability and age at disease onset in DM1 patients with variant repeats (Braida et al., 2010; Pešović et al., 2018) and with pure repeats (Morales et al., 2020) illustrate that variation in age at disease onset is modulated by variation in somatic instability.

DNA mismatch repair pathways (MMR) resolve unusual DNA–DNA and DNA–RNA structures formed during transcription. MMR can intensify intergenerational repeat instability and permit additional expansion in somatic cells (Castel et al., 2010; Reddy et al., 2014; Khristich and Mirkin, 2020; Malik et al., 2021). Most repeat expansion diseases displayed somatic instability, which creates variation in toxicity and repeat size across tissues in the same patient. Remarkably, somatic expansions are detected in terminally differentiated cells such as neurons and myofibers, makes it DNA replication independent process (Pearson et al., 1997; Gonitel et al., 2008; Keogh et al., 2017; Neil et al., 2021). The detailed mechanisms of somatic instability in repeat expansion diseases were recently reviewed (Khristich and Mirkin, 2020). Recent studies supported a role for somatic instability in disease pathogenesis implicating FAN1 and MMR proteins as modifiers of the age of onset in Huntington disease (Lee et al., 2015, 2017; Deshmukh A. et al., 2021b, Deshmukh et al., 2021 A. L.a; Porro et al., 2021) and spinocerebellar ataxias (SCAs) (Bettencourt et al., 2016), and alteration of MMR is sufficient to overturn somatic instability and can moderate toxicity in Huntington disease mice (Kovalenko et al., 2012; Pinto et al., 2013). In DM1, expansion-biased repeat instability in somatic cells is continuous throughout an individual’s life (Monckton et al., 1995; Wong et al., 1995), and is reflected to contribute directly to the progressive nature of the disease (Morales et al., 2012). The differential rates of somatic instability cause differences in the pathogenicity of repeat expansions across tissues. Somatic instability of the CTG repeat generates alleles in brain and skeletal muscle with repeat tracts considerably longer than in leukocytes (Ashizawa et al., 1993; Thornton et al., 1994), possibly aggravating RNA toxicity in those tissues.

## *De novo* mutation contributes to diseases

A lot has been found about the cause of *de novo* mutations, but how it contributes to disease is not well understood. There is no question that identifying the mechanism & the mutation load between tissues, specifically affected tissues, is going to help to understand how this contributes. For example, autism spectrum disorder (ASD) - a known and common form of neurodevelopmental intellectual disabilities (ID) described by a combination of deficits in communication and social interaction together with repetitive and restrictive behaviors (Kanner, 1968; Baio et al., 2018), is associated with *de novo* mutations*. ADNP* is one of the most frequently mutated genes in blood DNA through targeted molecular inversion probe sequencing studies and multiple recent whole-exome sequencing in ASD/ID cohorts (McRae et al., 2017; Stessman et al., 2017; Satterstrom et al., 2020). Nothing is known about how *de novo* mutations contribute to the extreme clinical variability of autism, a knowledge that would be beneficial in clinical assessment, diagnosis, and management.

*De novo* mutations are the most extreme form of rare genetic variation, have been subjected to less stringent evolutionary selection, more deleterious, on average, than inherited variation (Crow, 2000; Eyre-Walker and Keightley, 2007), which makes these mutations crucial candidates for causing genetic diseases that arise sporadically. A list of sporadic genetic disorders that are caused by *de novo* germline mutations are listed in the Table 1. Cancer is known to have somatic *de novo* mutations, which is covered elsewhere [for coverage of this topic, see reviews (Ding et al., 2010; Meyerson et al., 2010)]. Disorders caused by genetic mosaicism where reviewed elsewhere recently (Moog et al., 2020).

**TABLE 1:** List of genetic disorders caused by *de novo* mutations.

Disorders	Characteristics
Autism spectrum disorder (ASD) (O’Roak et al., 2011; O’Roak et al., 2012; Devlin et al., 2012; O’ Roak et al., 2012; Sanders et al., 2012; Iossifov et al., 2014)	Difficulties in social communications and interactions. Restricted, repetitive patterns of behavior, activities, or interests and sensory problems
Intellectual disability (ID) (De Vries et al., 2005; Vissers et al., 2010)	Certain limitations in mental abilities that affect cognitive functioning, communication skills, social and self-care skills
Schizophrenia (Girard et al., 2011; Xu et al., 2011)	Psychosis, apathy and withdrawal, and cognitive impairment, which cause problems in social and occupational functioning, and self-care
Down syndrome (Allen, 1974)	Child is born with an extra copy of their 21st chromosome—thus its other name is trisomy 21. This causes mental and physical developmental delays and disabilities
Schinzel–Giedion syndrome (Hoischen et al., 2010)	Skeletal dysplasia, congenital hydronephrosis, and severe developmental retardation
Kabuki syndrome (Ng et al., 2010)	Skeletal abnormalities, distinctive facial features and intellectual disabilities (ID)
Bohring–Opitz syndrome (Hoischen et al., 2011)	Multiple malformations, failure to thrive, facial anomalies and severe intellectual disabilities (ID)
Proteus syndrome (Lindhurst et al., 2011)	Mosaic or patchy overgrowth and hyperplasia of various organs and tissues
CHARGE syndrome (Vissers et al., 2004)	Arises during early fetal development and affects various organ systems, such as heart, eyes and ears
KBG syndrome (Sirmaci et al., 2011)	Facial dysmorphisms, macrodontia, skeletal anomalies and developmental delay
Baraitser–Winter syndrome (Rivière et al., 2012b)	Intellectual disability (ID) that ranges from mild to profound and typical craniofacial features
Microdeletion syndrome (Hassfurther et al., 2015; Kogan et al., 2015)	Mild to moderate intellectual disabilities, autism spectrum disorders, learning delays, or normal intelligence, epilepsy and mental illness
Coffin–Siris syndrome (Santen et al., 2012; Tsurusaki et al., 2012)	Abnormal head and facial (craniofacial) area, resulting in a coarse facial appearance
Adrenoleukodystrophy (Wang et al., 2011)	Failure of adrenal glands, progressive brain damage and, eventually, death
Crouzon syndrome (Toriello and Meck, 2008)	Premature fusion of the skull bones (craniosynostosis), exophthalmos, and midface hypoplasia
Multiple Endocrine Neoplasia Type 2 (Toriello and Meck, 2008)	Prevalence of medullary thyroid carcinoma and risk of developing other specific tumors that affect additional glands of the endocrine system
Charcot–Marie–Tooth disease type 1a (Pentao et al., 1992; Berg et al., 2010)	A rare genetic neurological disorder. It affects the peripheral nerves
Achondroplasia (Toriello and Meck, 2008)	Limited range of motion at the elbows, dwarfism, small fingers, large head size (macrocephaly), and normal intelligence
Apert Syndrome (Toriello and Meck, 2008)	Premature closing of cranial sutures. Certain fingers and toes fused or webbed
Duchenne muscular dystrophy (Helderman-van Den Enden et al., 2009)	Skeletal muscle weakness and degeneration
PIK3CA-related overgrowth spectrum (PROS) (Canaud et al., 2021)	Severe functional impairment, pain, vascular & neurological complications, seizures, and developmental delay, etc.
Paroxysmal nocturnal hemoglobinuria 1 (PNH1) (Li and Williams, 2013)	Hemoglobinuria, abdominal pain, smooth muscle dystonias, fatigue, and thrombosis
X-linked alpha-thalassemia mental retardation (Li and Williams, 2013)	Sometimes associated with myelodysplastic syndrome, with cases often associated with somatic mutations
Neurofibromatosis 1 (NF1) (Li and Williams, 2013)	Cafe-au-lait spots, Lisch nodules in the eye, and fibromatous tumors of the skin
Cardiac myocyte (Li and Williams, 2013)	Affect electrical communication and associate with a large minority of atrial fibrillation cases
Alport syndrome (Li and Williams, 2013)	X-linked dominant disorder characterized by kidney disease, hearing loss, and eye abnormalities
Lissencephaly, or smooth brain (Li and Williams, 2013)	Lethal in males, but milder forms have been associated with somatic mosaics in two patients with predominantly posterior subcortical band heterotopia
Autoimmune lymphoproliferative syndrome (ALPS) (Li and Williams, 2013)	A disease of benign lymphoproliferation, elevated immunoglobulins, plasma IL-10 and FAS-L, and accumulation of double-negative T cells

## Clinical consequences

The clinical consequences of mosaicism depend upon which chromosome is involved, and where and when a mutation arises. The identification of mosaicism is imperative in establishing a disease diagnosis, evaluating recurrence risk, and counseling. Mutation detection can be hampered by the presence of mosaicism in a patient. Both somatic and germline mosaicisms in humans have several clinical implications. Harmful *de novo* point mutations and indels that affect essential genes in development have been recognized as a prominent source of both common and rare genetic disorders (Vissers et al., 2010; O’Roak et al., 2011; De Ligt et al., 2012; Rauch et al., 2012; Allen et al., 2013; Iossifov et al., 2014; Chong et al., 2015). Somatic expansion of the *HTT* CAG repeat sequence is the rate-determining mechanism of HD (Lee et al., 2019). It is challenging to forecast the clinical consequences of mosaicism, as the patterns and distribution of abnormal cells can diverge widely depending on the timing of the mutation events. Depending on various factors, such as the degree of mosaicism and/or the gene involved, the clinical outcome of the mosaicism may be different in different disorders. For example, mosaicism of a specific chromosome may affect organ development, while mosaicism of other specific chromosome may affect muscular development. In addition to the chromosome, the prevalence or sheer numbers of the mosaic cell line are also matters. For an individual with a few mosaic cells, the effect of abnormal cells is masked by the number of normal cells. The opposite also true, for an individual with a large number of mosaic cells, the normal cells will be masked by the mosaic cells.

For unaffected parents who have an affected child and are planning a pregnancy, the recurrence risk may relate to the occurrence of new mutations at a particular gene or locus, the nature of mutational mechanism, the severity of the phenotype conferred by mosaicism, or the age and sex of the mosaic parent. For parents with germline mosaicism, the risk for a recurrence of another child with the disease is high. Mosaicism is also vital for disease mechanism. As an example, the protein kinase AKT1 with somatic mutations are correlated with Proteus syndrome, whereas mosaicism for post-zygotic mutations in genes for three essential components of the phosphatidylinositol 3-kinase (PI3K)–AKT signaling pathway that increase signaling can trigger a variety of related megalencephaly syndromes (Poduri et al., 2013). Compared to the same mutation present in a constitutional state, mosaic mutations can result in a less severe phenotype (Wallis et al., 1990), suggesting that more prevalent mosaicism is prone to have a more severe phenotype.

Most of the somatic mutations can be neutral and there has been speculation of how they could even be beneficial in some cases. For example, they may be contributing to functional diversity of neurons (Newman et al., 2017). Somatic mosaicism in neurons is generated mostly by *de novo* insertions of long interspersed nuclear elements (LINE retrotransposons) while undertaking the last neural progenitor divisions. As a population, neurons with low or high transposable elements insertions may express diverse subcategories of ion channels or neuronal adhesion molecules and thus have different firing properties. The origination of such a diverse neuronal population initiates the stochastic input required for network formation (Newman et al., 2017).

## Tools and techniques to detect mosaicism

Detection of mosaicism relies on applying subtle genotyping techniques that can detect low-level mosaicism in a more tedious fashion. Fluorescence *in situ* hybridization (FISH) have been used to analyze mosaic embryos. Molecular cytogenetic techniques (e.g., single-nucleotide polymorphism [SNP] array, array comparative genome hybridization [aCGH], chromosomal microarray analyses (CMA), quantitative polymerase chain reaction [qPCR], high-resolution next-generation sequencing (NGS) are better tools than FISH, because they can provide information on the copy number of all 24 types of chromosome (Munné and Wells, 2017). Mosaicism for primary trisomies in prenatal samples can be detected by quantitative fluorescent PCR (QF-PCR) and karyotype analysis (Donaghue et al., 2005). Non-invasive prenatal testing (NIPT) is used to detect confined placental mosaicism (CPM) (Eggenhuizen et al., 2021). Targeted strategies (e.g., droplet digital PCR) is a very powerful and efficient tool for sensitive detection and quantification of mosaicism (Mohiuddin et al., 2022).

## Conclusion and future perspectives

The identification of mosaic disorders is mounting day by day, which can be uniformly distributed throughout an organism, tissue-specific or segmental, and the germ line, somatic tissues or both can be affected. It can occur at numerous stages of development or adult life and can be triggered by mutation from a variant genotype to a normal genotype or vice versa.

The element of genetic counselling that addresses recurrence risks for patients with mosaic disorders is challenging. This is because genetic counselling necessitates analysis of sample cells within a given tissue and also because of the fact that mosaicism may be tissue-specific or tissue-limited. It is undoubtedly challenging to diagnose of tissue-limited mosaicism when the affected tissue is not skin or blood, tissues that are most frequently analyzed in clinical laboratories. Powerful genomic tools like NGS or targeted strategies (e.g., droplet digital PCR) might be useful for sensitive detection and quantification of mutations. These techniques will develop standard tools for the superior assessment of recurrence risks in families for which a genetic disease is triggered by *de novo* disease gene mutation event.
